# JAZF1 ameliorates age and diet-associated hepatic steatosis through SREBP-1c -dependent mechanism

**DOI:** 10.1038/s41419-018-0923-0

**Published:** 2018-08-28

**Authors:** Qin Wei, Baoyong Zhou, Gangyi Yang, Wenjing Hu, Lili Zhang, Rui Liu, Minyan Li, Kuan Wang, Harvest F. Gu, Youfei Guan, Zhiming Zhu, Hongting Zheng, Jun Peng, Ling Li

**Affiliations:** 1 Key Laboratory of Diagnostic Medicine (Ministry of Education) and Department of Clinical Biochemistry, College of Laboratory Medicine, Chongqing Medical University, Chongqing, P. R. China; 2 Department of Endocrinology, the Second Affiliated Hospital, Chongqing Medical University, Chongqing, P. R. China; 3 Department of Hepatobiliary Surgery, First Affiliated Hospital, Chongqing Medical University, Chongqing, P. R. China; 4 School of Basic Medicine and Clinical Pharmacy, China Pharmaceutical University, Nanjing, P. R. China; 5 Department of Clinical Science, Intervention and Technology, Karolinska University Hospital, Karolinska Institutet, Huddinge, Stockholm, Sweden; 6 Advanced Institute for Medical Sciences, Dalian Medical University, Liaoning, P. R. China; 7 Department of Hypertension and Endocrinology, Daping Hospital, Third Military Medical University, Chongqing Institute of Hypertension, Chongqing, P. R. China; 8 Department of Endocrinology, Xinqiao Hospital, Third Military Medical University, Chongqing, P. R. China

## Abstract

JAZF zinc finger 1 (JAZF1) is involved in glucose and lipid metabolisms. However, its role in aging- and nutrient-related hepatic steatosis is unclear. In the current study, we demonstrated that JAZF1 expression was markedly down-regulated in obesity-associated mice and nonalcoholic fatty liver disease (NAFLD) patients. During aging, JAZF1 expression was gradually down-regulated in both C57BL/6 J and JAZF1-Tg mice. In JAZF1-Tg mice, body fat content and hepatosteatosis were protected from HFD-induced steatosis, and accompanied by decreased lipogenesis gene expression. The inhibitory effects of hepatic steatosis in JAZF1-Tg mice, however, were disappeared during aging. In hepatocytes, over-expression of JAZF1 attenuated, while knockdown of JAZF1 enhanced the expression of lipogenesis genes. The over-expressing of JAZF1 in hepatocytes displayed the increased adenosine monophosphate-activated protein kinase (AMPK) phosphorylation and decreased sterol regulatory element-binding protein 1c (SREBP-1c) expression. The roles of JAZF1 were partially attenuated by Compound C. Mechanistically, JAZF1 suppressed SREBP-1c expression through the inhibition of transcriptional activity of liver X receptor response elements (LXREs) in the SREBP-1c promoter. Data illustrate that JAZF1 may have a crucial role in the regulation of age and nutrient-associated hepatosteatosis through an AMPK/SREBP-1c-dependent mechanism.

## Introduction

Aging is a complex process characterized by a general and irreversible decline in an organism’s fitness and capacity to survive. Aging is an important risk factor for all obesity-associated diseases, including type 2 diabetes mellitus (T2DM), cardiovascular disease (CVD), and nonalcoholic fatty liver disease (NAFLD)^1^. The complex regulatory networks, which are relative to aging and the pathogenesis of these diseases, need to be further elucidated.

NAFLD has been reported as one of the most common age-related diseases, impacting ~30% of all population^2^. Furthermore, approximately 25% NAFLD patients can progress to non-alcoholic steatohepatitis (NASH) and 10–15% patients with NASH develop hepatocellular carcinoma^3,4^. NAFLD is associated with insulin resistance (IR), T2DM and CVD. The incidence of T2DM and NAFLD has a younger trend and the question concerning the relationship between NAFLD and aging has been addressed^5,6^. However, aging and obesity have been generally considered as the most prevalent causes for NAFLD^7^. In addition, the mechanisms underlying the pathogenesis of NAFLD are still poorly understood. Therefore, investigation of the molecular mechanisms in aging and nutrient-associated NAFLD will provide useful information for identification of unique targets for therapeutic intervention of hepatosteatosis.

Juxtaposed with another zinc finger gene 1 (JAZF1) encodes a 27-kd nuclear protein containing 3 putative zinc finger motifs. This gene is highly expressed in fat and testes and widely in other tissues^8^. Six years ago, we reported that JAZF1 overexpression inhibited lipid formation and promoted lipolysis in vitro^9^. Later on, Jang et al. demonstrated that body weight, blood glucose and hepatic lipid accumulation in JAZF1-transgenic (JAZF1-Tg) mice fed a high fat diet (HFD) were lower than those of HFD-fed C57BL/6 J mice^10^. Furthermore, we found that JAZF1 overexpression protected against the development of atherosclerosis in apolipoproteinE knockout (ApoE KO) mice^11^ and attenuated the expression of pro-inflammatory cytokines in vivo and in vitro^12^. Recently, we reported that JAZF1 over-expression decreased hepatic glucose production and increased the phosphorylation of hepatic insulin signaling molecules via PI3-kinase/Akt-dependent manner^13^. Unfortunately, the absences of JAZF1 knock-out models due to embryonic lethality have severely hindered systematical functional study of JAZF1. Thereby, the impact of JAZF1 on age and nutrient-associated NAFLD and its molecular mechanisms are still remained unknown.

In the current study, we first generated JAZF1-Tg mice and then investigated the role of JAZF1 in age-associated and nutrient-induced hepatic steatosis. Data from our experiments may provide the first evidence to understand the molecular mechanisms of JAZF1 in aging and nutrient-induced hepatic steatosis with sterol regulatory element-binding protein 1c (SREBP-1c)-dependent pattern.

## Results

### Hepatic JAZF1 is down-regulated in human NAFLD and obesity-related mice

To investigate whether JAZF1 is involved in hepatic steatosis, we examined hepatic JAZF1 expression in db/db mice, C57BL/6 J fed with HFD and adiponectin knockout (Adipoq KO) mice, and patients with NAFLD. The mRNA and protein expression levels of JAZF1 were significantly reduced in the liver of db/db and Adipoq KO mice (Fig. S1A and B, Supporting information) compared to C57BL/6 J mice and also reduced in NAFLD patients (Fig. S1C and D) compared to control subjects. In addition, we established hepatic steatosis model induced by dietary fat over-loading in C57BL/6 J and Adipoq KO mice by feeding HFD for 12 weeks. As shown in Fig. S1A and B, HFD-fed mice had significantly decreased hepatic JAZF1 gene/protein expression compared to standard diet (SD)-fed mice. Data suggested a possible involvement of JAZF1 in both genetic- and environmental-related hepatic steatosis in animals and humans.

### Effect of aging on anthropometric and plasma parameters in SD-fed JAZF1-Tg mice

Metabolic parameters in SD-fed mice are summarized in Table S1. Body and liver weight, abdominal fat, fasting blood glucose (FBG), cholesterol, triglyceride, free fatty acid (FFA), alanine transaminase (ALT) and aspartate transaminase (AST) were markedly elevated in 2 + 6 and/or 2 + 12 month-old C57BL/6 J and JAZF1 mice when fed SD compare to 2 + 3 month-old littermates (*P* < 0.05 or *P* < 0.01). Body and liver weight, abdominal fat and FBG had a trend toward decrease in JAZF1-Tg mice compared to Wild Type (WT) mice, although statistical significance was not reached. When compared with 2 + 6 and 2 + 12-month-old C57BL/6 J mice, JAZF1-Tg mice aged by 2 + 6 and /or 2 + 12 months exhibited a marked decrease of total cholesterol (TC) and triglyceride (TG) in serum (Table S1). Low-density lipoprotein cholesterol (LDL-C) was also significantly decreased in 2 + 6-month-old JAZF1-Tg mice compared to age-matched WT mice (Table S1). In addition, ALT levels were significantly decreased in 2 + 6 and 2 + 12-month-old JAZF1-Tg mice in relation to age-matched WT littermates, whereas AST levels were decreased only in 2 + 12-month-old JAZF1-Tg mice (Table S1, *P* *<* 0.01).

### Decreased hepatic fat accumulation and expression of lipogenic enzymes in JAZF1- Tg mice during aging

**Fig. 1:**
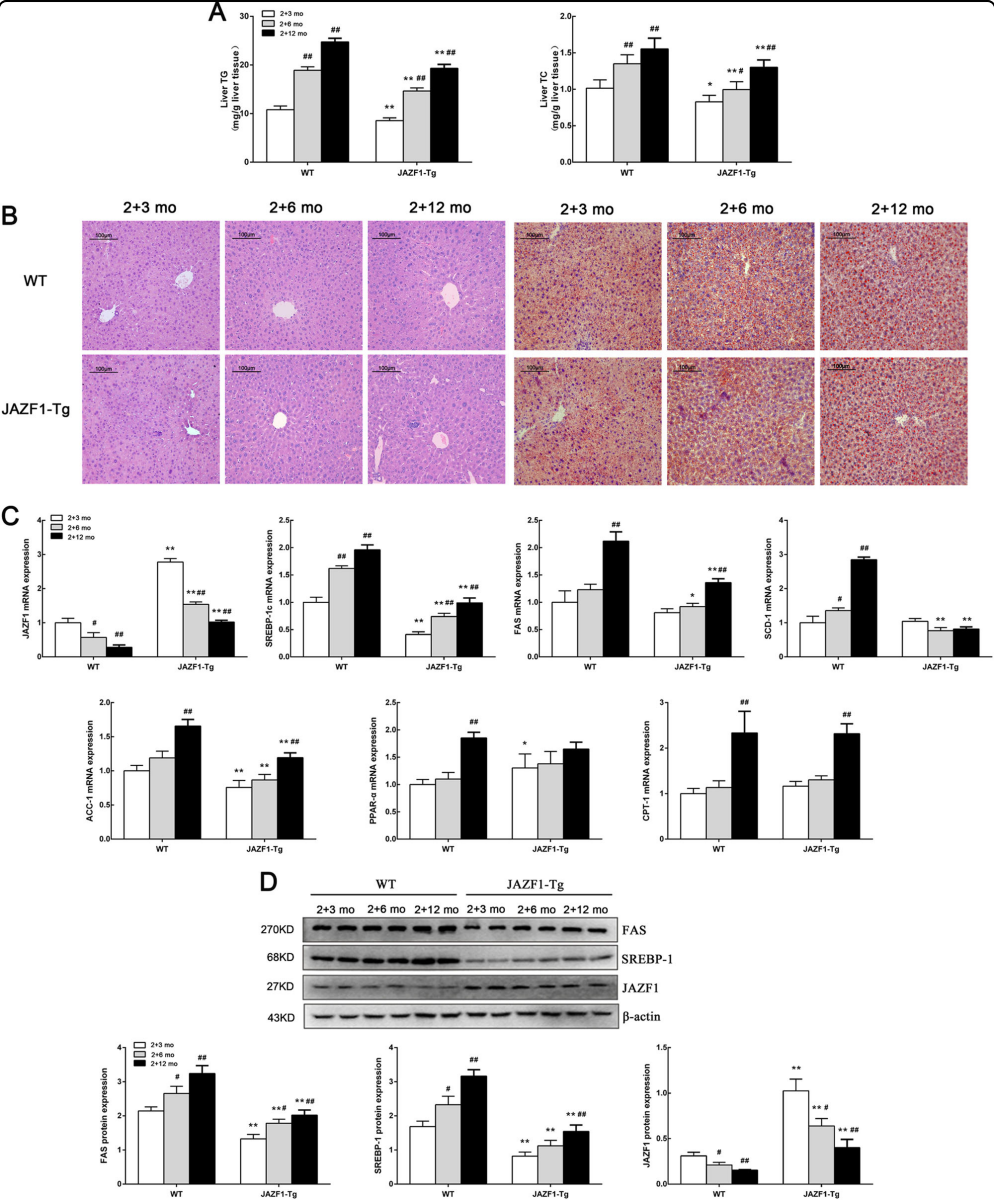
JAZF1 overexpression reduces hepatic lipogenesis during aging. **a** Liver triglyceride and cholesterol content in JAZF1-Tg and C57BL/6 J mice. **b** Staining of hematoxylin-eosin and Oil Red O in livers of JAZF1-Tg and C57BL/6 J mice. Representative images are shown (200×). **c** JAZF1 mRNA expression in the livers of JAZF1-Tg and WT mice during aging and the expression of genes related to fatty acid oxidation and lipogenesis in the livers of JAZF1-Tg and WT mice during aging. **d** The protein expression in the liver of JAZF1-Tg and control mice during aging. Data are expressed as mean ± SD. *P *<* 0.05; **P *<* 0.01 vs. age-matched WT mice, ^#^*P* *<* 0.05; ^##^*P* *<* 0.01, vs. 2 + 3-month-old mice. *n* = 5 for each group

As shown in Fig. 1a, hepatic TG and TC contents were gradually increased in both WT and JAZF1-Tg mice during aging (*P* *<* 0.05 or *P* *<* 0.01). When compared with aged-matched WT mice, TG and TC levels were lower in JAZF1-Tg mice (*P* *<* 0.05 or *P* *<* 0.01). In accordance with the decreased liver TG content, hematoxylin and eosin (H&E)-stained liver slides revealed less steatosis in SD-fed JAZF1-Tg mice compare to the WT littermates on the same diet during aging (Fig. 1b left). Oil Red O staining of the livers also showed reduced hepatic lipid deposition in JAZF1-Tg mice, whereas hepatic lipid deposition were significantly increased in both WT and JAZF1-Tg mice during aging (Fig. 1b right). To further investigate the cause of reduced lipid deposition in the liver of JAZF1-Tg mice during aging, the gene expressions of hepatic fatty acid oxidation and lipogenesis were measured in the liver of these mice. JAZF1 mRNA and protein expression gradually reduced in the liver of WT and JAZF1-Tg mice during aging (Figs. 1c, d). The mRNA expressions of fatty acid synthase (FAS), SREBP-1c, acetyl CoA carboxylase (ACC-1) and stearoyl CoA desaturase-1 (SCD-1) in JAZF1-Tg mice were significantly down-regulated compared with age-matched WT littermates, whereas carnitine palmitoyltransferase-1 (CPT-1) expression was not changed (Fig. 1c). However, the mRNA expression of peroxisome proliferators activator receptors alpha (PPAR-α) was up-regulated in 2 + 3-month-fed JAZF1-Tg mice only in relation to age-matched C57BL/6 J mice. The mRNA expressions of SREBP-1c, FAS, ACC-1, CPT-1 were gradually increased following aging in both C57BL/6 J and JAZF1-Tg mice, whereas SCD-1, PPAR-α expressions were significantly increase only in 2 + 12-month-fed WT mice (Fig. 1c). Western blot analyses showed that the protein levels of hepatic SREBP-1 and FAS were significantly decreased in JAZF1-Tg mice compared with age-matched WT mice (Fig. 1d). However, the protein levels of hepatic SREBP-1 and FAS were significantly increased during aging in both WT and JAZF1-Tg mice (Fig. 1d).

### Effect of aging on anthropometric and plasma parameters in JAZF1-Tg mice fed with HFD

To explore the pathological roles of JAZF1 over-expression in obese mice, we investigated the metabolic alterations in JAZF1-Tg and WT mice fed with a HFD during aging. Body and liver weight, abdominal fat, FBG, TG, TC, LDL-C and FFA were markedly decreased in JAZF1-Tg mice than in age-matched WT mice when fed HFD (Table S2). Both ALT and AST levels were also lower with aging in JAZF1-Tg mice compare with age-matched WT mice, consistent with attenuated liver injury and steatosis (Table S2). Body weight, liver weight, abdominal fat, FBG, TG, FFA, ALT and AST significantly increase as aging in both genotypes compared to 2 + 3-month-old littermates. LDL-C increases only in 2 + 12- month-fed compared to 2 + 3-month (*P* *<* 0.05), TC significantly increases in 2 + 12-month-fed WT mice and 2 + 6-month-fed JAZF1-Tg mice.

### Effect of aging on HFD-induced lipid accumulation in the liver of JAZF1-Tg mice

**Fig. 2:**
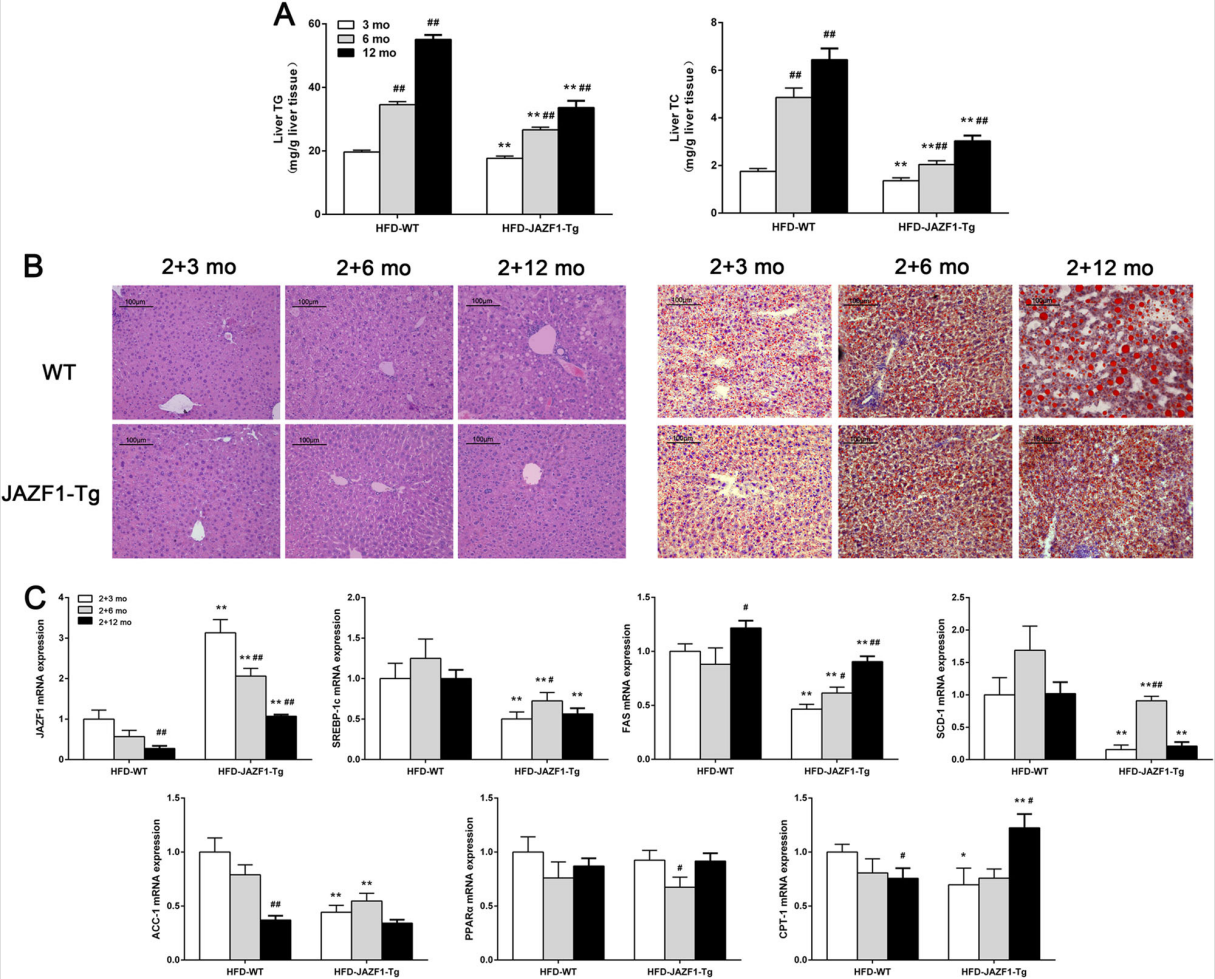
Effect of aging on HFD-induced hepatic fat accumulation in JAZF1-Tg and C57BL/6 J mice. **a** Liver triglyceride and cholesterol content in JAZF1-Tg and C57BL/6 J mice aged 5, 8, and 14 months. **b** H&E staining of liver sections (left) and oil red O staining of liver (right) (200×). **c** The mRNA expression of JAZF1 and genes related to fatty acid oxidation and lipogenesis in the livers of HFD-fed JAZF1-Tg and WT mice aged 5, 8, and 14 months. Data are expressed as mean ± SD. *P *<* 0.05; **P *<* 0.01 vs. age-matched WT; ^#^*P* *<* 0.05; ^##^*P* *<* 0.01 vs. 2 + 3-month-old mice. *n* = 5 for each group

**Fig. 3:**
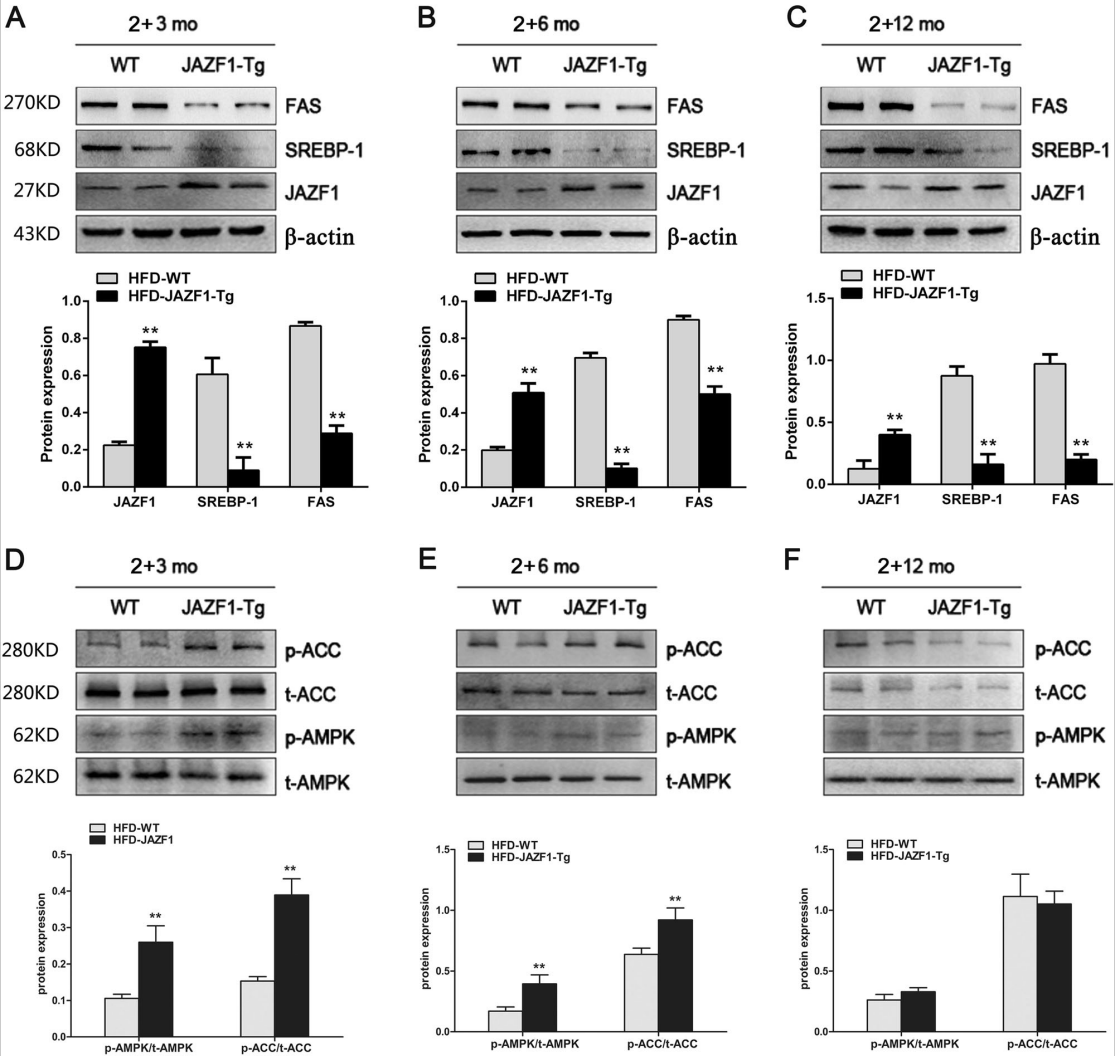
The protein expressions of lipogenic genes and the phosphorylation of AMPK and ACC in the liver of WT and Tg-JAZF1 mice fed an HFD. The protein expression of hepatic FAS and SREBP-1 in WT and Tg-JAZF1 mice aged 5 (**a**), 8 (**b**), and 14 (**c**) months. The phosphorylation of hepatic AMPK and ACC in WT and JAZF1-Tg mice aged 5 (**d**), 8 (**e)**, and 14 **(f)** months. Data are expressed as mean ± SD. **P *<* 0.01 vs. HFD-WT. *n* = 5 for each group

In both WT and JAZF1-Tg mice fed a HFD, hepatic TG and TC contents were gradually increased in both WT and JAZF1-Tg mice following aging (*P* *<* 0.01), whereas in HFD-fed JAZF1-Tg mice, TG and TC were lower than those of age-matched WT mice with HFD during aging (Fig. 2a, All *P* *<* 0.01). Results from H&E and Oil-Red-O staining of hepatic sections showed a reduced hepatic lipid deposition in JAZF1-Tg mice than in age-matched WT mice, when under a HFD. In both WT and JAZF1-Tg mice, hepatic lipid deposition was significantly increased following aging (Fig. 2b). To further explore the cause of JAZF1 over-expression-improved hepatic fat accumulation following aging, the gene expression of fatty acid oxidation and lipogenesis in liver were measured JAZF1-Tg and WT mice under a HFD for 3, 6 and 12 months. We found that JAZF1 expression were gradually decreased in both WT and JAZF1-Tg mice following aging,whereas in JAZF1-Tg mice, JAZF1 expression was still higher than in age-matched WT mice (Fig. 2c). Importantly, the expressions of fat storage-related gene in the liver, including SREBP-1c, SCD-1 and FAS, were significantly decreased in JAZF1-Tg mice than in age- matched WT mice during aging (Fig. 2c), whereas ACC-1 mRNA expression was also decreased in JAZF1-Tg animals aged 2 + 3 months (young) and 2 + 6 months (middle), but not 2 + 12 months (Fig. 2c). However, PPAR-α mRNA expression was unchanged in JAZF1-Tg mice with aging (Fig. 2c). CPT-1 mRNA expression in JAZF1-Tg mice was decreased at month 2 + 3, unchanged at month 2 + 6 and increased at month 2 + 12 (Fig. 2c). Western blot analyses showed the protein levels of hepatic SREBP-1 and FAS were markedly decreased in JAZF1-Tg mice compared to age-matched C57BL/6 J mice (Fig. 3a–c). Because NAFLD represents a spectrum of liver disease including simple fatty infiltration (steatosis), inflammation and cirrhosis, we thus examined some inflammatory and fibrotic markers. We found that under HFD feeding, mRNA expressions of interleukin-6 (IL-6), tumor necrosis factor-α (TNF-α), fibrotic genes collagen type III alpha 1(COL3A1) and Laminin in JAZF1-Tg mice were lower than in WT mice (Fig. S2A-D), suggesting that JAZF1 overexpression inhibits inflammatory reaction and liver fibrosis in vivo.

To investigate whether adenosine monophosphate (AMP)–activated protein kinase (AMPK) and ACC activation are required for the effect of JAZF1 on hepatic lipogenesis, we examined hepatic phosphorylation of AMPK and ACC in JAZF1-Tg and WT mice with various ages. We found that when fed a HFD, hepatic phosphorylation of AMPK and ACC was significantly increased in JAZF1-Tg mice aged from 2 + 3 (young) to 2 + 6 (middle) months compare to age-matched WT mice (Figs. 3d, e), whereas hepatic phosphorylation of AMPK and ACC showed no difference between JAZF1-Tg and WT mice aged 2 + 12 months (Fig. 3f).

### Effects of JAZF1 on lipid accumulation and lipogenic gene expression in vitro

**Fig. 4:**
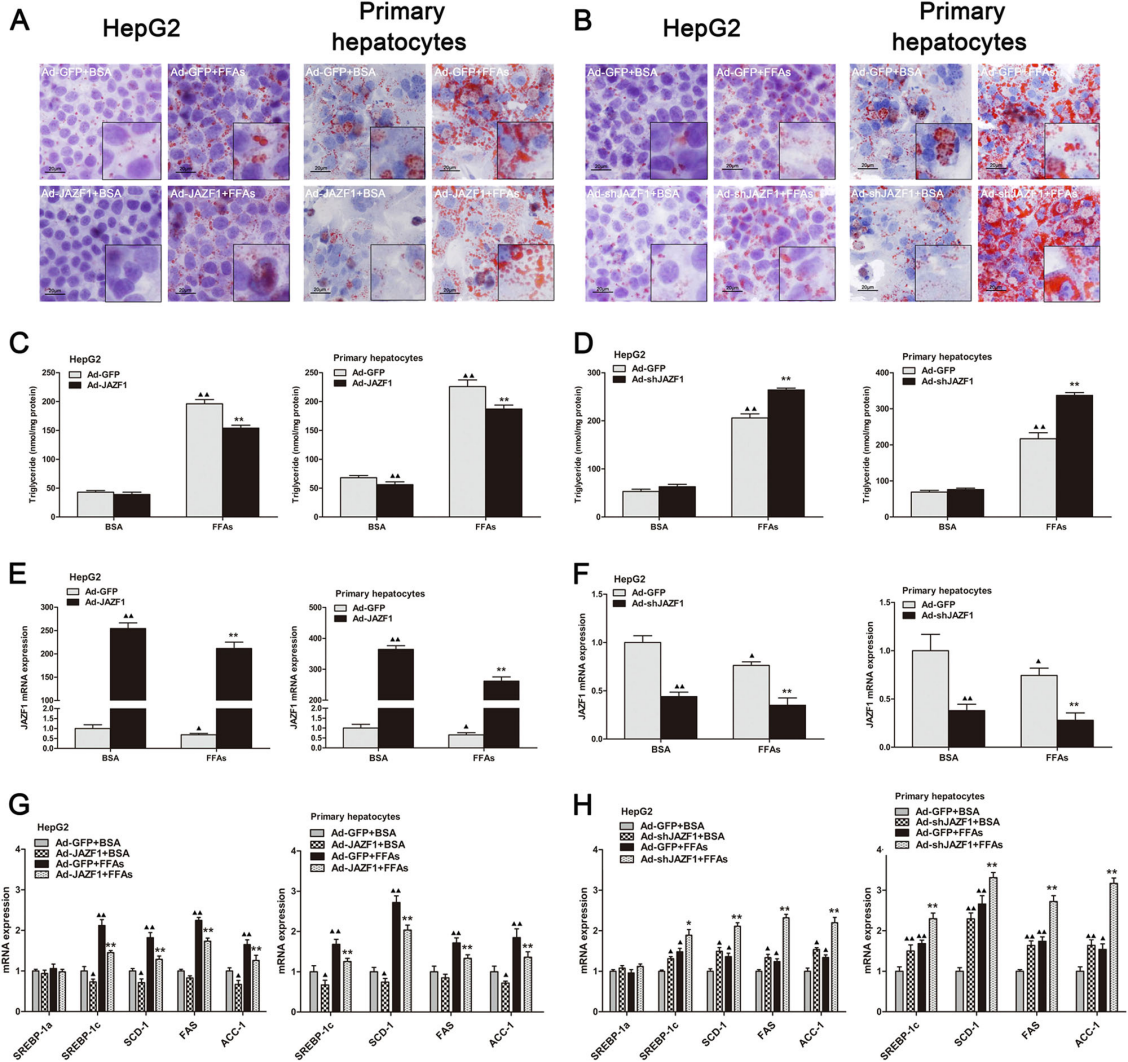
The effects of JAZF1 on hepatic TG accumulation in vitro. Oil Red O staining of HepG2 cells and primary hepatocytes treated with FFAs or BSA after transfection of Ad-GFP and Ad-JAZF1 (**a**) or Ad-*sh*JAZF1 (**b**) (200×). The illustration was magnified by 2 times. Triglyceride contents in HepG2 cells and primary hepatocytes (**c–d**) treated as in (**a–b**). JAZF1 mRNA expression in HepG2 cells and primary hepatocytes (**e–f**) treated as in (**a–b**). The mRNA expression of lipogenic genes in HepG2 cells and primary hepatocytes **(g–h**) treated as in (**a–b**). The data are expressed as the mean ± SD and are representative of at least three independent experiments. *P < 0.05; **P *<* 0.01 vs. Ad-GFP + FFAs; ^▲^*P* < 0.05; ^▲▲^*P* < 0.01, vs. Ad-GFP + BSA

**Fig. 5:**
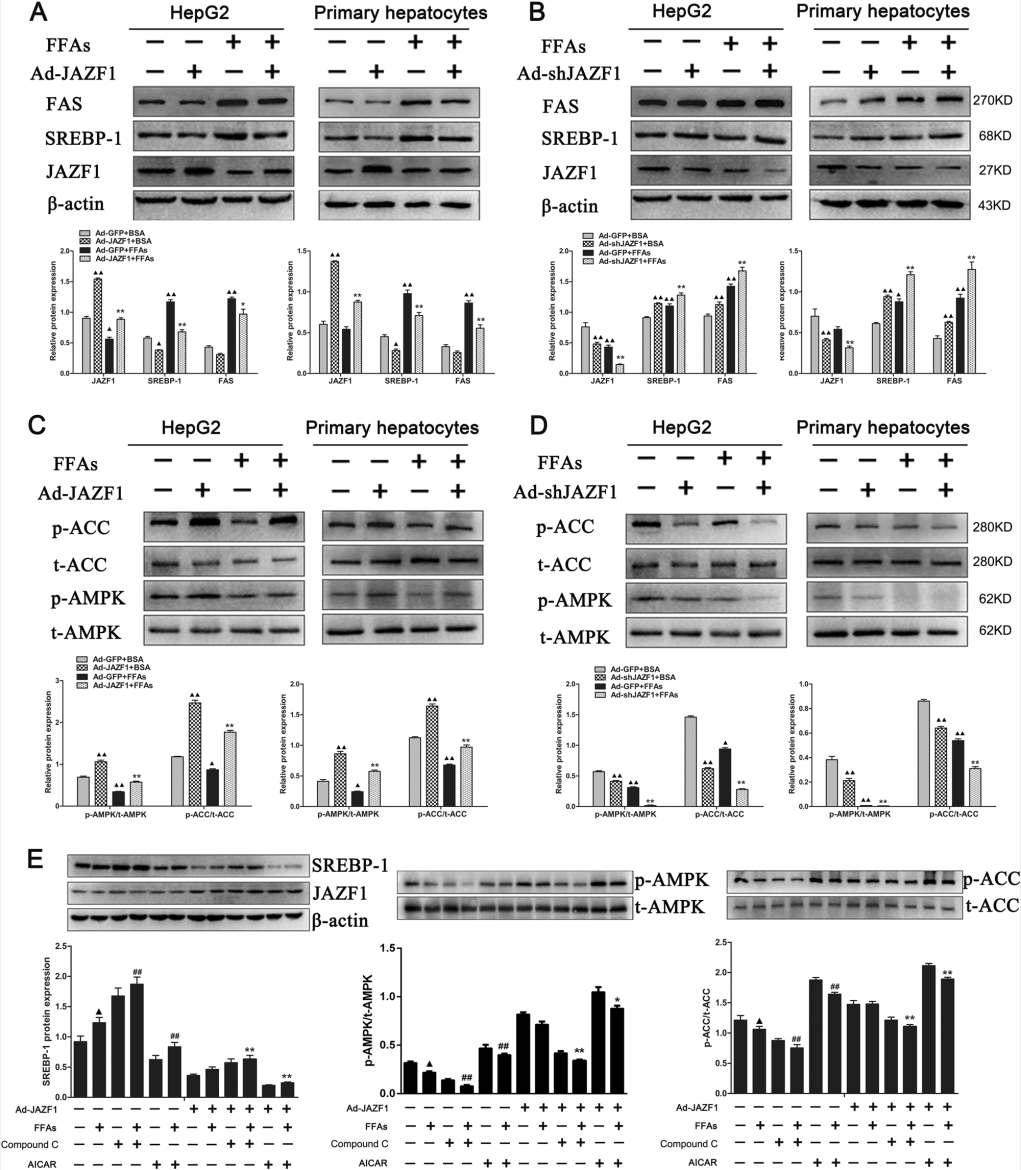
The effects of JAZF1 on the protein expressions of lipogenic genes and phosphorylation of AMPK and ACC in vitro. The FAS, SREBP-1 and JAZF1 protein levels in HepG2 cells and primary hepatocytes treated with or without FFAs after transfection of Ad-GFP and Ad-JAZF1 **(a)** or Ad-*sh*JAZF1 **(b)**. The phosphorylation of AMPK and ACC in HepG2 cells and primary hepatocytes treated as in (**a**–**b**) after transfection of Ad-GFP and Ad-JAZF1 (**c**) or Ad-*sh*JAZF1 (**d**). *P *<* 0.05; **P *<* 0.01 *vs*. Ad-GFP + FFAs; ^▲^ *P* < 0.05; ^▲▲^*P* < 0.01 vs. Ad-GFP + BSA. **e** HepG2 cells were transfected with Ad-JAZF1 for 36 h. After treatment with AICAR, Compound C or DMSO alone as control, cells were incubated with FFAs for 24 h. SREBP-1 protein (left), AMPK (middle) and ACC (right) phosphorylation were analyzed by Western blots. ^▲^ *P* < 0.05, vs. blank controls; ^#^*P* *<* 0.05; ^##^*P* *<* 0.01 vs. FFAs treatment; *P *<* 0.05 and **P *<* 0.01 vs. Ad-JAZF1 + FFAs. Data (means ± SD) are representative of results in at least three independent experiments

To further determine the role of JAZF1 on hepatic steatosis, Adenovirus expressing JAZF1 (Ad-JAZF1) and small hairpin RNA directed against the coding region of JAZF1 (Ad-*sh*JAZF1) were constructed for up- or down-regulation of JAZF1 in HepG2 cells and Mouse primary hepatocytes (MPHs). As depicted in Fig. 4a, Oil Red O staining demonstrated that decreased lipid stores were presented in HepG2 cells and MPHs when cells were treated with Ad-JAZF1 and FFAs, compared to adenovirus encoding green fluorescence protein (Ad-GFP) and FFAs treatment. In contrast, Ad-*sh*JAZF1 treatment demonstrated more lipid droplets in HepG2 cells and MPHs, compare with the control group (Fig. 4b). In parallel, the cellular TG content was significantly decreased in Ad-JAZF1 plus FFAs-treated HepG2 cells and MPHs compare to Ad-GFP and FFAs treatment (Fig. 4c), whereas Ad-*sh*JAZF1 treatment led to an increase of TG content in both HepG2 cells and MPHs treated with FFAs (Fig. 4d). To explore the cause of JAZF1-induced reduction in hepatic fat accumulation in vitro, we demonstrated the expression of JAZF1 and genes relative to fatty acid oxidation and lipogenesis in HepG2 cells and MPHs. As expected, JAZF1 mRNA/protein expression was significantly up-regulated in Ad-JAZF1 treated MPHs and HepG2 cells (Figs. 4e and 5a), whereas Ad-*sh*JAZF1 treatment led to reduction of JAZF1 expression in these cells (Figs. 4f and 5b). In addition, mRNA expression of the fat storage-related genes, SREBP-1c, FAS, SCD-1and ACC-1 was significantly decreased in Ad-JAZF1 treated MPHs and HepG2 cells (Fig. 4g), whereas Ad-*sh*JAZF1 treatment led to increasing mRNA expression in these genes (Fig. 4h). Western blot analyses showed the protein levels of SREBP-1 and FAS were also decreased in Ad-JAZF1 treated cells (Fig. 5a) and increased in Ad-*sh*JAZF1-treated cells (Fig. 5b) compared to the controls. Importantly, in MPHs and HepG2 cells treated with Bovine Serum Albumin (BSA) or FFAs, Ad-JAZF1 resulted in a significant increase in AMPK and ACC phosphorylation (Fig. 5c). In contrast, Ad-*sh*JAZF1 treatment led to a significant reduction in the phosphorylation of AMPK and ACC in these cells (Fig. 5d).

### JAZF1 suppresses lipogenic gene expression via AMPK activation

To ascertain whether AMPK pathways are involved in the JAZF1-induced suppression of lipogenic genes, HepG2 cells were transfected using Ad-JAZF1 or Ad-GFP and then treated with Compound C (AMPK inhibitor) or AICAR (AMPK activator) in the absence or presence of FFAs. As expected, p-AMPK and p-ACC levels were obviously increased in the Ad-JAZF1 or AICAR-treated cells exposed to FFAs, whereas SREBP-1 was obviously inhibited in these cells (Fig. 5e). These results suggested that the effects of JAZF1 on AMPK and ACC phosphorylation, as well as on SREBP-1 expression were similar to the effects observed in AICAR-treated cells. Importantly, the ability of JAZF1 to phosphorylate AMPK and ACC and to inhibit SREBP-1 expression was diminished by Compound C (Fig. 5e), while further aggravated by AICAR. These results indicated that JAZF1 up-regulated ACC phosphorylation and down-regulated SREBP-1 expression in an AMPK-dependent manner.

### JAZF1 regulates SREBP-1c transcription by AMPK

**Fig. 6:**
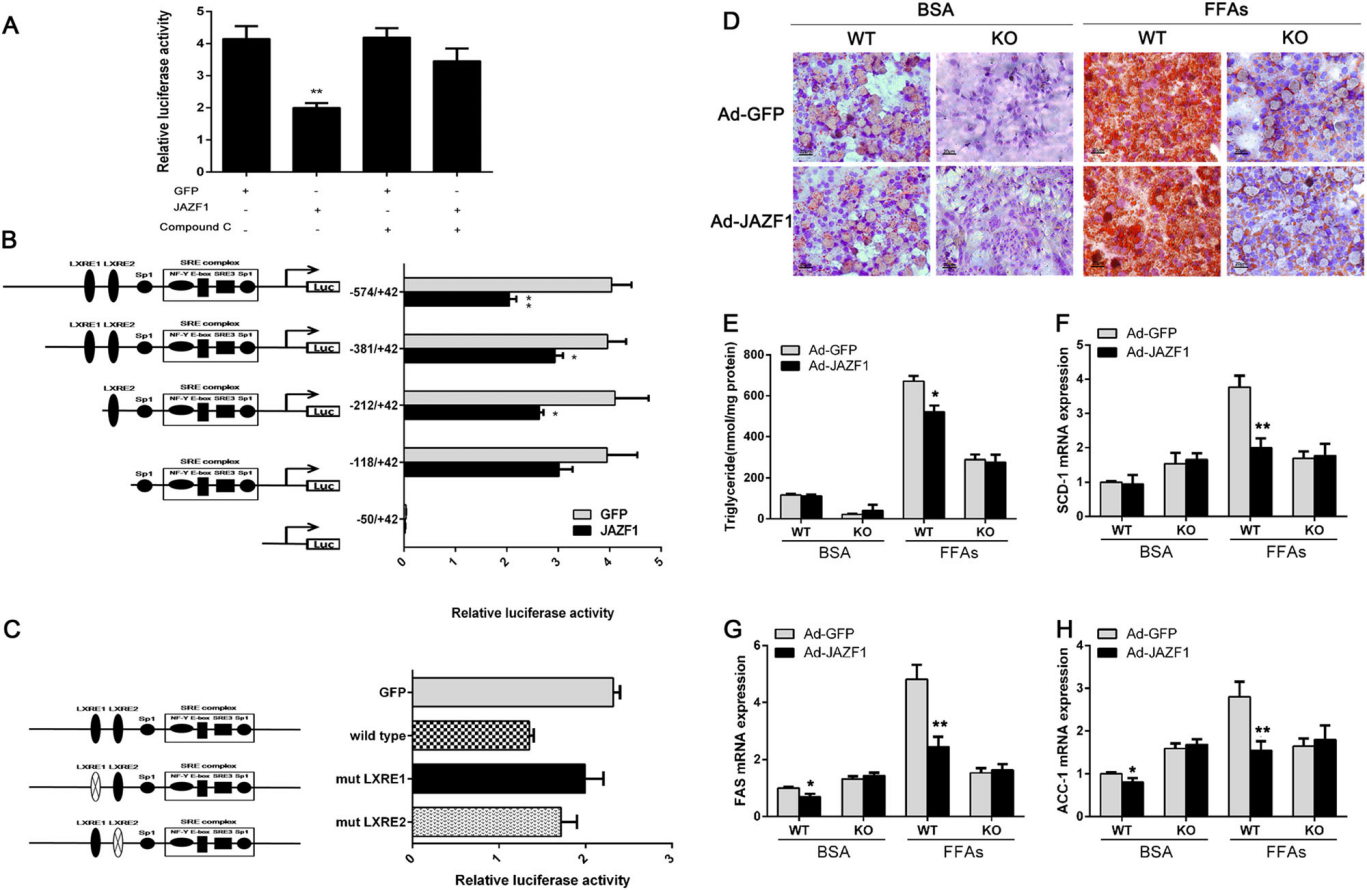
Transcriptional Regulation of SREBP-1c promoter by JAZF1 via AMPK pathway. **a** JAZF1-mediated transcriptional repression of the full-length SREBP-1c promoter (−574/ + 42 bp) in HepG2 cells. **b** Effects of JAZF1 overexpression on SREBP-1c promoter activities of various deletion. **c** Site-directed mutagenesis analysis. Schematic diagram of the positions of the LXREs within the SREBP-1c promoter and design of deletion constructs (Left). HepG2 cells were transfected with a plasmid containing wild-type (−574/ + 42 bp) or one of the mutated SREBP-1c gene promoters (LXRE1 mut or LXRE2 mut, LXRE1/LXRE2 mut) together with JAZF1 or GFP overexpression plasmids and pRL-SV40. Renilla luciferase activities were measured after 48 h incubation of transfected hepatocytes (Right). Normalized luciferase activities are expressed as the means ± SD. *P *<* 0.05; **P *<* 0.01 vs. GFP. **d** Oil Red O staining of MPHs isolated from WT mice and SREBP-1c KO mice. MPHs were treated with BSA or FFAs after transfection of Ad-GFP and Ad-JAZF1 (200×). **e** Triglyceride contents in WT and SREBP-1c KO hepatocytes treated as in (**d**) after transfection. The mRNA expression of SCD-1 (**f**), FAS (**g**), and ACC-1 (**h**) in WT and SREBP-1c KO hepatocytes treated as in (**d**) after transfection. Data (means ± SD) are representative of results in at least three independent experiments. *P *<* 0.05; **P *<* 0.01 vs. Ad-GFP

To investigate the molecular mechanism of JAZF1 modulation of SREBP-1c expression, we then studied the impact of JAZF1 on the SREBP-1c transcriptional activity in HepG2 cells. Dual luciferase reporter assay showed that the transcriptional activity of SREBP-1c promoter was significantly suppressed by pIRES2-JAZF1, whereas dorsomorphin (Compound C), an AMPK inhibitor, completely reversed the inhibition (Fig. 6a).

To determine the promoter region responsible for the JAZF1 regulation of SREBP-1c expression, we constructed various 5’deletions of the SREBP-1c promoter plasmid [pGL3-SREBP-1c (−574/ + 42)-luc]. The dual luciferase assay showed that the transcriptional activity of the JAZF1 regulated SREBP-1c promoter was significantly suppressed in pGL3-SREBP-1c (−574), pGL3-SREBP-1c (−381) and pGL3-SREBP-1c (−212), but not in pGL3-SREBP- 1c (−118) and pGL3-SREBP-1c (−50) (Fig. 6b). This finding suggested that a potential JAZF1-regulating site of the SREBP-1c promoter might be located in 574 to −118 bp upstream of transcriptional start site.

### Liver X receptor response elements (LXREs) is required for transcriptional regulation of JAZF1 on SREBP-1c promoter

Several studies demonstrated that SREBP-1c promoter contains two putative LXREs located at nucleotides −381/−212 and −212/−118, and one sterol regulatory element complex (SREs), the Nuclear fator Y(NF-Y), E-box, SRE3, and Sp1 sites located at −118/−50^14^. To further elucidate the site functionally responsible for the negatively transcriptional regulation of the SREBP-1c promoter by JAZF1, we mutated LXRE1 (−234/−218) or LXRE2 (−184/−168) element from the SREBP-1c luciferase reporter. In transiently transfected HepG2 cells, mutation of the LXRE1 (−234/−218) led to a 55.3% increase of the response to JAZF1, whereas loss of the proximal LXRE2 (−184/−168) led to a 27.1% increase (Fig. 6c). Therefore, data suggested that the LXREs within SREBP-1c promoter might play an important role in mediating the JAZF1-regulation of SREBP-1c gene transcription.

### JAZF1 over-expression does not suppress lipid accumulation and lipogenic gene expression in the MPHs of SREBP-1c KO mice

To further investigate the relationship between JAZF1 and SREBP-1c, we examined the influences of JAZF1 on fat accumulation and lipogenic gene expression in the MPHs of SREBP-1c KO mice. Lipid accumulation and TG contents in FFAs-treated MPHs from WT mice were significantly diminished by Ad-JAZF1 treatment, but not in the MPHs from SREBP-1c KO mice (Figs. 6d, e). Importantly, the mRNA expression of fat storage-related genes, FAS, SCD-1 and ACC-1 was significantly decreased by Ad-JAZF1 in FFAs-treated MPHs from WT mice, but unchanged in FFAs-treated MPHs from SREBP-1c KO mice (Figs. 6f–h). These data suggested a possible involvement of SREBP-1c in the effects of JAZF1 on hepatic lipid metabolism

## Discussion

In the current study, we employed HFD diet with a prolonged feeding for one year to establish a model of obesity with hepatic steatosis in WT and JAZF1-Tg mice, which mimics human NAFLD to explore the role of JAZF1 in age and HFD-related NAFLD in vivo. We found that genetic over-expression of JAZF1 attenuated many of the adverse metabolic features observed in HFD-fed WT mice. In JAZF1-Tg mice, selective effects were also noted with a decrease in hepatic fat accumulation, serum ALT and AST levels, and a specific decrease in the expression of genes related de novo lipogenesis compared to WT mice during aging, especially when fed with a HFD, these changes were more pronounced. This implicated that JAZF1-Tg mice became lower fat in liver by aging and HFD. The ‘adaptations’ to aging and HFD likely protected the liver from the development of liver injury and inflammation. Moreover, we utilized cell-based assays to demonstrate the role of JAZF1 on lipid metabolism, which might circumvent JAZF1 actions in other tissues. To overcome the potential shortcomings of HepG2 cells, we used mouse primary hepatocytes and human HepG2 to rigorously demonstrate the effects of JAZF1 on lipogenesis. In the agreement with observations in vivo, we found that over-expression of JAZF1 mediated by adenovirus attenuated FFAs-induced fat accumulation in both HepG2 cells and MPHs. Accompanying the decrease in lipid accumulation, the expression of lipogenesis genes, including SREBP-1c, FAS, SCD-1, and ACC-1, in HepG2 cells and MPHs was down-regulated. However, similar to other study^15^, the effects of treatment on lipogenetic gene expression between HepG2 cells and MPHs seem to be different, but no statistically significant. Possible reasons for this phenomenon may be related to abnormal metabolic phenotype and poor sensitivity to fatty acid synthesis in HepG2 cells^16,17^. Taken together, data from both in vivo and in vitro experiments clearly indicated that JAZF1 signaling was a potential process in hepatic steatosis occurring during aging and nutrient excess.

AMPK is a multi-subunit enzyme. A few years ago, we reported that JAZF1 had an important role in the regulation of energy equilibrium, insulin sensitivity and these events derived AMPK activation^13^. However, AMPK is also known as an important regulator of lipid biosynthetic pathways due to its phosphorylation and inactivation with key enzymes such as ACC and FAS^18–21^. Zhou *et al*. have reported that AMPK activation suppresses SREBP-1, an important transcription factor that regulates fatty acid, TG and cholesterol synthesis in liver^22^. In the current study, we found that overexpression of JAZF1 increased the phosphorylation of AMPK and ACC (inactivation), a rate-limiting enzyme in de novo lipogenesis, and inhibited SREBP-1 expression in vitro. Mechanistic studies led to the discovery that incubation of Ad-JAZF1-treated MPHs and HepG2 cells with AICAR, an AMPK activator, further increased ACC phosphorylation and suppressed SREBP-1 expression, whereas Compound C, a classical AMPK inhibitor, abolished JAZF1-mediated inhibition of SREBP-1 expression and increase of ACC phosphorylation. Concerning that the mechanism of JAZF1 activation of AMPK is currently unclear, we thus postulate that JAZF1 may directly regulate AMPK phosphorylation or indirectly regulates AMPK activity by modulating upstream signals. Further investigation to better understand the mechanism has been taken into our consideration.

Additionally, in MPHs from SREBP-1c KO mice, Ad-JAZF1 treatment did not show any effect for increased lipid stores induced by FFAs. These findings further implicate that SREBP-1c plays a critical role in JAZF1-mediated regulation of hepatic lipogenesis. Hence, we could speculate that JAZF1 inhibits the activation of lipogenic genes through AMPK/SREBP-1c pathway. Moreover, it has been reported that there is decreased signaling through PI3-kinase and Akt, and increased activity of SREBP-1c in the liver of *ob/ob* or HFD-fed mice during the IR state^23^. Therefore, inhibition of SREBP-1c expression by JAZF1 may be due to improved IR in vivo as well^13^.

To better understand the molecular mechanism by which JAZF1 suppresses SREBP-1c expression, we provide evidence that the transcriptional activity of SREBP-1c promoter is regulated by JAZF1 in HepG2 cells using dual luciferase assays. The AMPK inhibitor, Compound C, completely reverses the response of SREBP-1c promoter to JAZF1. The data are consistent with a mechanism by which the effect of JAZF1 on the transcriptional activity of SREBP-1c is mediated by AMPK. Furthermore, the detailed deletion and mutation analysis of SREBP-1c promoter revealed that the region of the SREBP-1c promoter responsible for JAZF1 activation was located within −574 to −118 bp upstream of the transcriptional start site, whereas the inactivation of SREBP-1c promoter by JAZF1 was partly abolished when the LXRE1 or LXRE2 was deleted. Based on these results, we concluded that JAZF1 negatively regulates mouse SREBP-1c promoter by AMPK in HepG2 cells. Therefore, it may be reasonable to consider that JAZF1 overexpression could cause a transcriptional abrogation of SREBP-1c promoter by AMPK, which in turn inhibits gene transcription involved in fatty acid and triglyceride synthesis, such as FAS^24,25^, thus contributing to the ameliorated steatosis in Ad-JAZF1- treated hepatocytes and in the liver of JAZF1-Tg mice [Fig. S3].

There are a couple of limitations in the current study. First, we had no JAZF1 knockout mice due to embryonic lethality. Second, considering the following study for other insulin target tissues such as muscle and fat and exploring the role of JAZF1 between these tissues, we didn’t constructed hepatic-specific transgenic mice. Third, the potential mechanisms for the down-regulation of hepatic JAZF1 in obese mice and NAFLD patients remain to be determined. Nevertheless, as a pilot study, data from our experiments are of interest to reveal the potential link between JAZF1 and hepatic steatosis.

In summary, the present study for the first time provides evidence that hepatic JAZF1 (gene/protein) expression is decreased in patients and mice with NAFLD, while JAZF1-Tg mice are protected against age-related and HFD-induced obesity and hepatic steatosis. Furthermore, we demonstrate that JAZF1 may act as a transcriptional co-repressor, depending on AMPK binding to LXREs of SREBP-1c promoter and inhibits SREBP-1c transcription, which in turn represses the expression of genes involved hepatic lipid synthesis. Thereby, JAZF1 may be a novel target for treatment of aging- and diet-related NAFLD.

### Experimental procedures

Animal preparation and human liver tissues, C57BL/6 J, db/db and Adipoq KO mice, respectively, were purchased from Animal Centers of Chongqing Medical University and Shanghai Biomodel or Ganismsci & Tech Develop CO., Ltd. Shanghai, China. SREBP-1c KO mice were purchased from the Jackson Laboratory (stock#-004365). Seven week–old Male mice were adapted to the environment for 1 week before experiments. JAZF1-Tg and SREBP-1c KO mice were generated as previously described^26^. C57BL/6 J, SREBP-1c KO, Adipoq KO and db/db mice (8 weeks old) were fed with either standard diet (SD; 10% fat) or high-fat diet (HFD; 45% fat; Medicine Inc. Jiangsu, China) for 12 weeks. In age-related metabolic study, C57BL/6 J and JAZF1-Tg mice (*n* = 30 each) at 8 weeks old were randomly divided into six groups and fed with either SD or a HFD for 12 weeks, 24 weeks or 48 weeks, respectively. Five mice of each group were sacrificed at weeks 12, 24, and 48 after being placed on the respective diet. Blood samples were collected for metabolic parameter measurements. Tissues were harvested for future analyses. The human liver tissues were collected under percutaneous liver biopsy from 10 patients with NAFLD and 10 donors of liver transplantation from Department of Surgery, the First Affiliated Hospital, Chongqing Medical University or Xinqiao Hospital, Third Military Medical University. The diagnosis of NAFLD was based on diagnostic criteria from the Asia-Pacific regional guidelines and biopsy^27^. The patient’s information is listed in Table S3. The protocols were reviewed and supported by the Ethics Committee of the Chongqing Medical University, China.

### Construction and purification of recombinant adenovirus vectors

Ad-JAZF1 and Ad-*sh*JAZF1 were generated using the AdEasy Adenoviral Vector System (Qbiogene) or the pAdxsi system (SinoGenoMax Co. Ltd, Beijing, China), and adenovirus expressing green fluorescent proteins (Ad-GFP) as a control was constructed as previously described^9,11,12^. Large-scale amplification and purification of adenoviruses were performed with the ViraBind Adenovirus Purification Kit (Cell Biolabs, San Diego, USA), and recombinant adenoviruses were stored at −80 °C.

### Cell culture and treatment

MPHs were isolated from WT mice, JAZF1-Tg mice or SREBP-1c KO mice aged 8 weeks as previously described^28^. MPHs were cultured in DMEM/F12. HepG2 cells were cultured in DMEM with 10% FBS. When cells reached 70% confluence, they were transfected with Ad-JAZF1 or Ad-*sh*JAZF1 or Ad-GFP for 36 h, and then exposed to 1 mM FFAs mixture or 1% BSA as a control in serum free medium for 20 h. The solutions of oleate acid (50 mM) and palmitate (50 mM) were dissolved in isopropanol and FFAs mixture (1 mM) at a 2:1 ratio of oleate/palmitate was used to induce fat-overloading as previously reported^29^. For AMPK pathway studies, HepG2 cells were treated with AICAR, an AMPK activator, (0.5 mmol/L, Sigma-Aldrich, St. Louis, MO, USA) or Compound C, an AMPK inhibitor, (20 μmol/L, Calbiochem, Darmstadt, Germany) or DMSO as a vehicle for 60 min before FFAs treatment.

### Histological examination and cell Oil Red O staining

Formalin-fixed liver and adipose tissues from human or mice were processed, and paraffin sections (5 µm) were stained with H&E. Frozen liver sections, HepG2 cells and MPHs on a 6-well plate were stained with 0.15% oil red O according to standard procedures.

### Determination of cholesterol and triglyceride contents

TG and total TC contents were measured and expressed as micrograms of lipid in per milligram of cellular protein or per gram of tissue weight, using commercial kit (Applygen Technologies Inc., Beijing, China) according to the manufacturer’s protocol^30^.

### Transfections and dual luciferase assay

The DNA fragments, which contained the region from −574 to + 42 bp of the mouse SREBP-1c promoter, and all the deletion constructs (−381/ + 42 bp, −212/ + 42 bp, −118/ + 42 bp and −50/ + 42 bp) were generated by genomic PCR as previously described^31^. Purified PCR products and pGL3-basic luciferase reporter vector (promega, WI, USA) were digested with Xhol and HindIII. Recombinant plasmids (pGL3-SREBP-1c-luc) were verified by restriction endonuclease digestion and by sequencing the DNA. The primers used for the DNA fragments were listed in Table S4. Mutations in pSREBP-1c (−574/ + 42 bp)-luc, described previously by Deng et al^32^, were created by site-directed mutagenesis using the Quik Change kit (Stratagene, La Jolla, CA, USA). The sequences of primers used to generate site-directed mutations were listed in Table S5. For the dual luciferase assay, HepG2 cells were seeded into 12-well plates and transfected with 1.0 μg/well reporter plasmid, 0.04 μg/well of Renilla-luc plasmid (pRL-SV40, Promega), and 0.8 μg/well of pIRES2- JAZF1 or pIRES2-GFP using Lipofectamine 2000 (Invitrogen, Carlsbad, CA, USA), as previously described^9^. After transfection, the cells were incubated in the presence or absence of Compound C and cultured in DMEM with 10% FBS. Forty-eight hours after transfection, luciferase activity was estimated using the Dual- Luciferase ®Reporter Assay System (Promega).

### Protein and mRNA analysis

Real-time quantitative PCR was performed as described previously^11^. The primer pairs are listed in Table S6. Protein analysis was performed with western blots as described previously^11^. Primary antibodies included anti-SREBP-1 (sc-8984, Santa Cruz Biotechnology, INC. Dallas, TX, USA), anti-FAS (FAS, sc-55580, Santa Cruz), anti-ACC (3662 S, Cell Signaling, Danvers, MA, USA), anti-phospho-ACC (Ser79, 3661 S, Cell Signaling), anti-AMPK (2532 S, Cell Signaling), anti-phospho- AMPK (2535 T,Cell Signaling), anti- JAZF1 (ab80329, Abcam, Cambridge, MA, USA) and anti-β-actin (Research Diagnostics Inc, Beijing, China).

### Statistical analysis

Data are presented as means ± SD. Statistical analysis was performed in Microsoft Excel and Prism software (GraphPad, La Jolla, CA, USA). Statistical significance was evaluated using the two-tailed unpaired Student’s *t* test and among more than two groups by one-way analysis of variance. *P*-value < 0.05 was considered statistically significant.

## Electronic supplementary material

Supplemental Material

Supplementary Figure Legends
