# Supplementary material for: JAZF1 ameliorates age and diet-associated hepatic steatosis through SREBP-1c -dependent mechanism
Source: Cell Death Dis. 2018 Aug 28;9(9):859. doi: 10.1038/s41419-018-0923-0 (PMC6113258; doi:10.1038/s41419-018-0923-0)
Supplement: Supplementary file 1 — Supplemental Material [file 41419_2018_923_MOESM1_ESM.docx]

***Supplemental documents:***

**JAZF1 ameliorates** **age- and diet-associated hepatic steatosis through SREBP-1c–dependent mechanism**

*Running title: JAZF1 regulates lipid metabolism*

Qin Wei^1#^, Baoyong Zhou^3#^, Gangyi Yang^2^, Wenjing Hu^1^, Lily Zhang^1^, Rui Liu^1^, Minyan Li^2^,Kuan Wang^1^, [Harvest F. Gu](http://www.ncbi.nlm.nih.gov/pubmed/?term=Gu%20HF%5Bauth%5D)^4,5^, Youfei Guan^6^, Zhiming Zhu^7^, Hongting Zheng^8^, Jun Peng^6^, Ling Li^1*^,

**Fig. S1 Hepatic JAZF1 is down-regulated in human NAFLD and obesity-related mice**


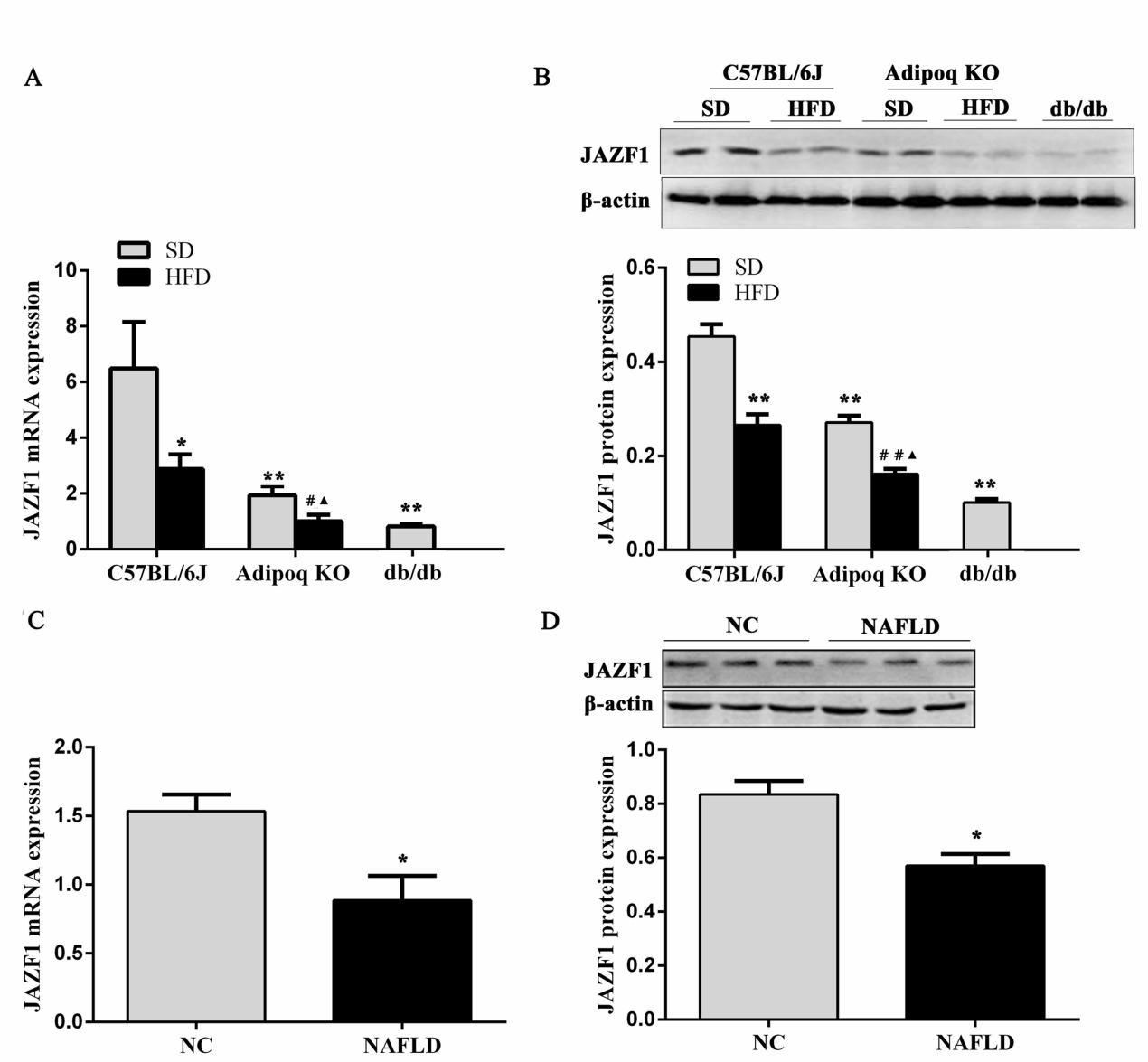


27KD

43KD

43KD

27KD

JAZF1 mRNA (A) and protein (B) expression levels in the liver of SD or HFD-fed C57BL/6J, Adipoq KO and db/db mice (n = 5 for each group). JAZF1 mRNA(C) and protein (D) expression in the liver of NAFLD patients and healthy subjects (n =10 for each group). Data are expressed as mean ± SD. **P* < 0.05, ***P* < 0.01 compared with SD-fed C57BL/6J mice or healthy subjects. ^#^*P* < 0.05,^##^ *P* < 0.01 compared with SD-fed ADI KO mice. ^▲^*P* <0.01 compared with HFD-fed C57BL/6J mice.

**Fig. S2 HFD-induced inflammation and fibrosis are attenuated in JAZF1-Tg mice**

**
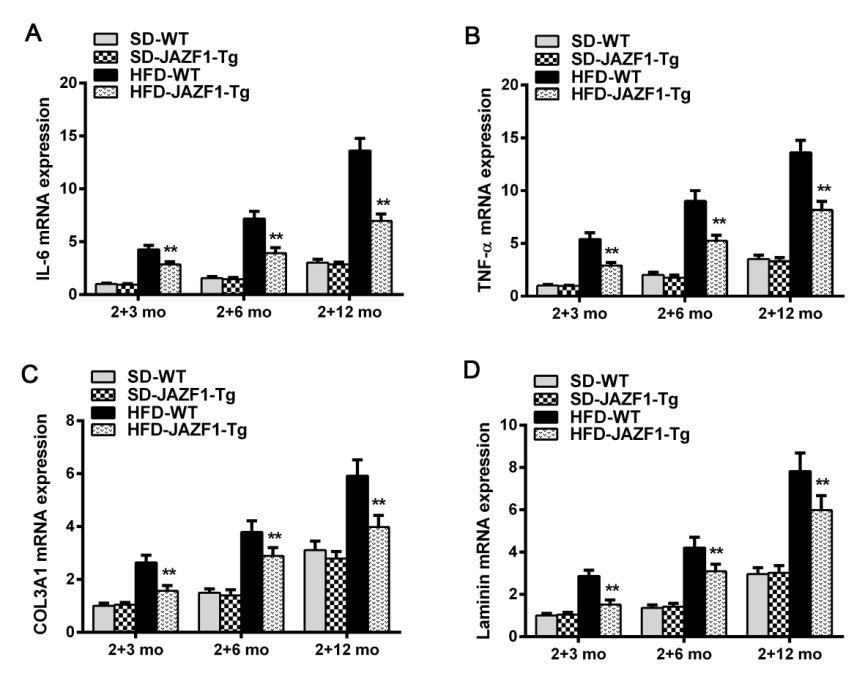
**

Data are presented as the means ± SD and demonstrate mRNA expression levels of the genes encoding for IL-6 **(A)**, TNFα **(B)**, COL3A1**(C)** and Laminin **(D)** in the liver of WT and JAZF1-Tg mice fed with SD or HFD as indicated (n=3); ** *P* <0.01 *vs.* HFD-WT mice.

**Fig. S3 Schematic diagram for the mechanism of JAZF1 in the inhibition of** **hepatosteatosis**


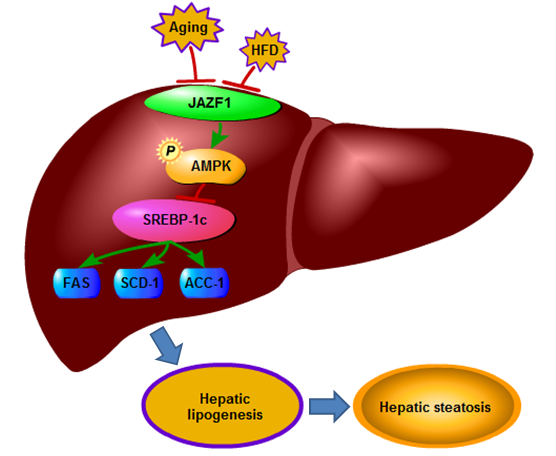


**Table S1 Metabolic parameters in SD-fed animals**

| Parameters | WT | |  | | JAZF1-Tg | |
| --- | --- | --- | --- | --- | --- | --- |
|  | 2+3 mo | 2+6 mo | 2+12 mo | 2+3 mo | 2+6 mo | 2+12 mo |
| Body weight (g) | 29.9 ± 0.5 | 31.8 ± 0.5^##^ | 31.9 ± 0.6^##^ | 29.0 ± 0.3 | 31.5 ± 0.1^##^ | 31.5 ± 0.2^##^ |
| Liver weight (g) | 1.2 ± 0.06 | 1.3 ± 0.08^#^ | 1.4 ± 0.05^##^ | 1.2 ± 0.04 | 1.3 ± 0.09^##^ | 1.3 ± 0.05^##^ |
| Abdominal fat (g) | 0.6 ± 0.05 | 0.8 ± 0.2^#^ | 0.8 ± 0.08^##^ | 0.5 ± 0.05 | 0.7 ± 0.1^#^ | 0.7 ± 0.08^#^ |
| FBG (mmol/L) | 5.8 ± 0.7 | 6.8 ± 0.9 | 9.1 ± 0.8^##^ | 5.7 ± 0.8 | 6.2 ± 1.5 | 8.7 ± 0.8^##^ |
| Cholesterol (mmol/L) | 2.8 ± 0.4 | 3.4 ± 0.2^#^ | 3.7 ± 0.5^#^ | 2.7 ± 0.4 | 3.2 ± 0.5 | 3.1 ± 0.5* |
| Triglyceride (mmol/L) | 0.5 ± 0.06 | 0.7 ± 0.1^##^ | 0.8 ± 0.2^##^ | 0.5 ± 0.08 | 0.5 ± 0.1* | 0.7 ± 0.1*^#^ |
| FFA (mmol/L) | 0.5 ± 0.07 | 0.9 ± 0.1^##^ | 0.7 ± 0.2^#^ | 0.4 ± 0.1 | 0.9 ± 0.1^##^ | 0.6 ± 0.2^#^ |
| HDL-C (mmol/L) | 1.1 ± 0.2 | 1.3 ± 0.4 | 1.2 ± 0.2 | 1.1 ± 0.3 | 1.1 ± 0.2 | 1.1 ± 0.3 |
| LDL-C (mmol/L) | 0.2 ± 0.03 | 0.3 ± 0.1 | 0.3 ± 0.06 | 0.2 ± 0.05 | 0.1 ± 0.05** | 0.2 ± 0.03^#^ |
| ALT (U/L) | 31.6 ± 5.0 | 67.4 ± 5.3^##^ | 86.7 ± 15.1^##^ | 30.2 ± 4.1 | 45.4± 4.9**^##^ | 52.8 ± 11.9**^##^ |
| AST (U/L) | 57.7 ± 9.3 | 69.6 ± 4.8^#^ | 105.0 ± 11.9^##^ | 46.6 ± 7.0 | 66.7 ± 6.2^##^ | 83.4 ± 11.7**^##^ |

SD, standard diet; FBG, fasting blood glucose; FFA, free fatty acid; HDL-C, high-density lipoprotein cholesterol; LDL-C, low-density lipoprotein cholesterol; ALT, Alanine transaminase; AST, aspartate transaminase. Data are presented as means ± SD. **P* < 0.05, ***P* < 0.01 *vs*. age-matched WT mice; ^#^*P* < 0.05,^##^*P* < 0.01 *vs*. 2+3 month-fed mice. (n = 5 for each group)

**Table S2 Metabolic parameters in HFD-fed animals**

| Metabolic parameters | WT | |  | | JAZF1-Tg | |
| --- | --- | --- | --- | --- | --- | --- |
|  | 2+3 mo | 2+6 mo | 2+12 mo | 2+3 mo | 2+6 mo | 2+12 mo |
| Body weight (g) | 33.1 ± 0.4 | 39.7 ± 0.6^##^ | 52.4 ± 0.9^##^ | 30.7 ± 0.2** | 33.1 ± 0.3**^##^ | 39.0 ± 1.2**^##^ |
| Liver weight (g) | 1.4 ± 0.03 | 1.5 ± 0.07^##^ | 2.0 ± 0.1^##^ | 1.3 ± 0.05** | 1.4 ± 0.1**^#^ | 1.5 ± 0.07**^##^ |
| Abdominal fat (g) | 1.3 ± 0.1 | 2.0 ± 0.3^##^ | 3.8 ± 0.5^##^ | 0.8 ± 0.08** | 1.1 ± 0.2**^#^ | 1.9 ± 0.3**^##^ |
| FBG (mmol/L) | 8.7 ± 0.8 | 10.8 ± 1.8^#^ | 17.5 ± 1.7^##^ | 7.0 ± 0.8** | 7.4 ± 0.7** | 11.6 ± 1.9**^##^ |
| Cholesterol (mmol/L) | 4.4 ± 0.7 | 5.3 ± 0.7 | 5.5 ± 0.8^#^ | 3.6 ± 0.5* | 4.2 ± 0.1**^#^ | 3.9 ± 0.3** |
| Triglyceride (mmol/L) | 0.8 ± 0.2 | 1.1 ± 0.2^##^ | 1.4 ± 0.2^##^ | 0.7 ± 0.2 | 0.9 ± 0.1** | 0.9 ± 0.1**^#^ |
| FFA (mmol/L) | 0.8 ± 0.1 | 1.3 ± 0.1^##^ | 1.4 ± 0.3^##^ | 0.7 ± 0.08 | 1.1 ± 0.1*^##^ | 1.1 ± 0.3*^#^ |
| HDL-C (mmol/L) | 1.5 ± 0.3 | 2.2 ± 0.5^#^ | 1.9 ± 0.2^#^ | 2.0 ± 0.2** | 1.4 ± 0.4**^#^ | 1.5 ± 0.2*^##^ |
| LDL-C (mol/L) | 0.3 ± 0.06 | 0.4 ± 0.1 | 0.5 ± 0.1^#^ | 0.2 ± 0.05* | 0.2 ± 0.04** | 0.3 ± 0.08*^#^ |
| ALT (U/L) | 52.4 ± 9.1 | 165.1 ± 10.3^##^ | 242.3 ± 62.7^##^ | 38.8 ± 7.4** | 58.9 ± 7.4**^##^ | 122.1 ± 19.8**^##^ |
| AST (U/L) | 79.2 ± 9.1 | 172.5 ± 14.2^##^ | 273.6 ± 74.5^##^ | 53.4 ± 7.5**^##^ | 81.5 ± 9.1**^##^ | 114.8 ± 15.6**^##^ |

HFD, high fat diet; FBG, fasting blood glucose; FFA, free fatty acid; HDL-C, high-density lipoprotein cholesterol; LDL-C, low-density lipoprotein cholesterol; ALT, Alanine transaminase; AST, aspartate transaminase. Data are presented as means ± SD. n = 5 for each group. * *P* < 0.05, ***P* < 0.01 *vs*. age-matched WT mice, ^#^*P* < 0.05, ^##^*P* < 0.01, *vs*. 2+3 month-fed mice.

**Table S3 Clinical and metabolic features of subjects with JAZF1 expression analysis**

| Control (N=10) NAFLD (N=10) |
| --- |
| Age (years) 52.0 ± 14.6 54.1 ± 11.7  BMI (kg/m^2^) 20.8 ± 2.8 24.7 ± 3.6*  ALT (U/L) 33.3 ± 21.6 46.2 ± 23.0*  AST (U/L) 28.7 ± 12.4 34.9 ± 13.3*  ALP (U/L) 114.6 ± 41.3 100.3 ± 50.6  Grade of steatosis  Grade 0 10 (100%)  Grade 1 6 (60%)  Grade 2 4 (40%) |

Data are expressed as mean ± SD. **P* < 0.05, ***P* < 0.01. BMI, body mass index; ALT, Alanine transaminase; AST, aspartate transaminase; ALP, Alkaline phosphatase.

**Table S4 Primers used for SREBP-1c promoter recombinant plasmids**

| **Promoter regions** | **Primers** |
| --- | --- |
| -574/+42 F | 5' -CCGCTCGAGGGATCCAGAACTGGATCATCAGCCCC-3' |
| R | 5' -CCCAAGCTTCCTAGGGCGTGCAGACGCTACCCCG-3' |
| -381/+42 F | 5' -CCGCTCGAGAAATGGTCCAGGCAAGTTCTGGGTG-3' |
| R | 5' -CCCAAGCTTCCTAGGGCGTGCAGACGCTACCCCG-3' |
| -212/+42 F | 5' -CCGCTCGAGGCGCTGGCGCAGACGCGGTTAAAGG-3' |
| R | 5' -CCCAAGCTTCCTAGGGCGTGCAGACGCTACCCCG-3' |
| -118/+42 F | 5' -CCGCTCGAGTGGGCGGGGCCCTAATGGGGCGCGG-3' |
| R | 5' -CCCAAGCTTCCTAGGGCGTGCAGACGCTACCCCG-3' |
| -50/+42 F | 5' -CCGCTCGAGCACGGAGGCGATCGGCGGGCTTTA-3' |
| R | 5' -CCCAAGCTTCCTAGGGCGTGCAGACGCTACCCCG-3' |

**Table S5 Primers used for site-directed mutation**

| **Plasmid** | **Primers** |
| --- | --- |
| mut LXRE1 F | 5'-GGGTTGGGACGACAGCCCGGGATCCACCAATCAGCGCGCGCTGGCG-3' |
| R | 5' -CGCCAGCGCGCGCTGATTGGTGGATCCCGGGCTGTCGTCCCAAC-3' |
| mut LXRE2 F | 5'-GCGGTTAAAGGCGGACCCGGGATCCACCAATCGGCCCCATTCAGAGC-3' |
| R | 5' -GCTCTGAATGGGGCCGATTGGTGGATCCCGGGTCCGCCTTTAACC-3' |

**Table S6 Specific primers used for RT-PCR analyses**

| **Gene Species Primer Sequence** |
| --- |
| JAZF1 Mus musculus F 5'-CCACTCCACCTCGACATA-3'  R 5'-CCACCTCTTCCTCATCATAC-3'  SREBP-1c Mus musculus F 5'-CAAGGCCATCGACTACATCCG-3'  R 5'-CACCACTTCGGGTTTCATGC-3'  SCD-1 Mus musculus F 5'-TGATGTTCCAGAGGAGGTA-3'  R 5'-CCAGAGTGTATCGCAAGAA-3'  PPAR-α Mus musculus F 5'-TGCCTTAGAACTGGATGAC-3'  R 5'-ATCTGGATGGTTGCTCTG-3'  ACC-1 Mus musculus F 5'-AGCAGTTACACCACATACAT-3'  R 5'-GTCATCACCATCTTCATTACC-3'  FAS  Mus musculus F 5'-CTCATCCACTCAGGTTCAG-3'  R 5'-AGGTATGCTCGCTTCTCT-3'  CPT-1 Mus musculus F 5'- AGCCAGACGAAGAACATC-3'  R 5'-CCTTGACCATAGCCATCC-3'  IL-6 Mus musculus F 5'- TAGTCCTTCCTACCCCAATTTCC-3'  R 5'- TTGGTCCTTAGCCACTCCTTC-3'  TNF-α Mus musculus F 5'- GATCGGTCCCCAAAGGGATG-3'  R 5'- TGAGGGTCTGGGCCATAGAA-3'  COL3A1 Mus musculus F 5'- CCCACAGCCTTCTACACCT-3'  R 5'- CCCATTCCTCCCACTCC-3'  Laminin Mus musculus F 5'- TCCGTACTACCTCTAAGAAT-3'  R 5'- GATAGCCACCCACATAA-3'  β-actin Mus musculus F 5'-AAGACCTCTATGCCAACAC-3'  R 5'-CTGCTTGCTGATCCACAT-3'  JAZF1 Homo sapiens F 5'-CGAGTATGACGAGGAGGA-3'  R 5'-GTTCTGTGACCATTCTTAGC-3'  SREBP-1a Homo sapiens F 5'-TCAGCGAGGCGGCTTTGGAGCAG-3'  R 5'-CATGTCTTCGATGTCGGTCAG-3'  SREBP-1c Homo sapiens F 5'-GGAGCCATGGATTGCACTTT-3'  R 5'-TCAAATAGGCCAGGGAAGTCA-3'  FAS Homo sapiens F 5'-GTCCACCAGCAACATCAG-3'  R 5'-TTCTCCAGCAAGCCATCT-3'  SCD-1 Homo sapiens F 5'-CGATATGCTGTGGTGCTTA-3'  R 5'-AAGGAGTGGTGGTAGTTGT-3'  ACC-1 Homo sapiens F 5'-AACCACATCTTCCTCAACTT-3'  R 5'-ACTTCCATACCGCATTACC-3'  β-actin Homo sapiens F 5'-AGACCTCTATGCCAACACAGT-3'  R 5'-TCGTACTCCTGCTTGCTGAT-3' |
