# Supplementary material for: JAZF1 ameliorates age and diet-associated hepatic steatosis through SREBP-1c -dependent mechanism
Source: Cell Death Dis. 2018 Aug 28;9(9):859. doi: 10.1038/s41419-018-0923-0 (PMC6113258; doi:10.1038/s41419-018-0923-0)
Supplement: Supplementary file 2 — Supplementary Figure Legends [file 41419_2018_923_MOESM2_ESM.docx]

**Supplementary Figure Legends:**

**Fig. S1 Hepatic JAZF1 is down-regulated in human NAFLD and obesity-related mice** JAZF1 mRNA (A) and protein (B) expression levels in the liver of SD or HFD-fed C57BL/6J, Adipoq KO and db/db mice (n = 5 for each group). JAZF1 mRNA(C) and protein (D) expression in the liver of NAFLD patients and healthy subjects (n =10 for each group). Data are expressed as mean ± SD. **P* < 0.05, ***P* < 0.01 compared with SD-fed C57BL/6J mice or healthy subjects. ^#^*P* < 0.05,^##^ *P* < 0.01 compared with SD-fed ADI KO mice. ^▲^*P* <0.01 compared with HFD-fed C57BL/6J mice.

**Fig. S2 HFD-induced inflammation and fibrosis are attenuated in JAZF1-Tg mice**

Data are presented as the means ± SD and demonstrate mRNA expression levels of the genes encoding for IL-6 **(A)**, TNFα **(B)**, COL3A1**(C)** and Laminin **(D)** in the liver of WT and JAZF1-Tg mice fed with SD or HFD as indicated (n=3); ** *P* <0.01 *vs.* HFD-WT mice.

**Fig. S3 Schematic diagram for the mechanism of JAZF1 in the inhibition of** **hepatosteatosis**

**Table S1.** Metabolic parameters in SD-fed animals.

**Table S2.** Metabolic parameters in HFD-fed animals.

**Table S3.** Clinical and metabolic features of subjects with JAZF1 expression analysis.

**Table S4.** Primers used for SREBP-1c promoter recombinant plasmids.

**Table S5.** Primers used for site-directed mutation.

**Table S6.** The specific primers sequence used for RT-PCR analysis.
